# Process and outcome evaluation of a social norms approach intervention on alcohol use among Flemish university students: a quasi-experimental study

**DOI:** 10.1186/s13690-024-01265-w

**Published:** 2024-03-28

**Authors:** Hanna van Roozendaal, Katleen Derickx, Koen Ponnet, Benedicte Deforche, Annelies Thienpondt, Inge Glazemakers, Stijn Verhulst, Jozef De Dooy, Nico van der Lely, Guido Van Hal

**Affiliations:** 1 Faculty of Medicine and Health Sciences, University of Antwerp, Wilrijk, Belgium; 2 Department of Communication Sciences, Faculty of Political and Social Sciences, Ghent University, Ghent, Belgium; 3 Department of Public Health and Primary Care, Faculty of Medicine and Health Sciences, Ghent University, Ghent, Belgium; 4 Department of Movement and Sport Sciences, Faculty of Physical Education and Physiotherapy, Vrije Universiteit Brussel, Brussels, Belgium; 5 University Centre for Child and Adolescent Psychiatry (ZNA-UKJA), Antwerp, Belgium; 6 Department of Paediatrics, Antwerp University Hospital, Edegem, Belgium; 7 Department of Paediatrics, Reinier de Graaf Hospital, Delft, Netherlands

**Keywords:** Students, Alcohol Drinking, Alcohol Drinking in College, Social Norms, Social Norms Approach, Social Media, Health Promotion, Campaign, Outcome and Process Assessment, Belgium

## Abstract

**Background:**

Alcohol consumption is prevalent among students, with a common tendency to overestimate peers' alcohol use, contributing to increased consumption. This misperception is evident among Flemish students. This study aimed to develop and assess a Social Norms Approach (SNA) intervention targeting Flemish students to correct misperceptions and subsequently reduce alcohol use.

**Methods:**

The 'Alcoholfacts' social media campaign was implemented using a quasi-experimental design from November 2022 to March 2023. A process evaluation followed Medical Research Council guidance, and intervention effects were evaluated using baseline and post-intervention surveys. Multiple linear regression with a Difference-in-Difference approach was performed for outcome assessment, using an intention-to-treat approach.

**Results:**

The process evaluation showed that 36.3% of the intervention group had seen the campaign and that most of the exposed students found the campaign credible (73.3%). However, 54.8% of the exposed students did not find the campaign appealing. Results of the outcome assessment indicated that students of the intervention group at endline estimated students’ alcohol consumption significantly lower (bootstrapped *p* = 0.013; B = -1.93, bootstrapped CI = -3.620 to -0.565) compared to students of the control group. However, no significant intervention effect on student’s alcohol consumption was found (bootstrapped *p* = 0.741; B = -0.32, bootstrapped CI = -2.101 to 1.534).

**Conclusions:**

The study supports the efficacy of an SNA campaign in correcting misperceptions but did not yield an immediate reduction in alcohol consumption. Future research should involve the target group in campaign material development to enhance attractiveness and impact.

**Supplementary Information:**

The online version contains supplementary material available at 10.1186/s13690-024-01265-w.

**Table:** 

Text box 1. Contributions to literature
• The discrepancy between students’ perception of alcohol consumption of peers and their actual alcohol consumption among Flemish students indicates the potential benefit of a Social Norms Approach (SNA) intervention.
• This study is the first in Belgium to implement and evaluate an SNA intervention targeting alcohol consumption and misperceptions of alcohol consumption of peers among university students.
• The study's findings support the efficacy of an SNA campaign in correcting misperceptions of alcohol consumption among students.
• Results of the process evaluation of this study might support other universities in developing similar SNA campaigns for students in the future.

## Background

Alcohol use is a major public health concern, with a total of 3.0 million deaths and 131.4 million disability-adjusted life-years caused by alcohol use worldwide [1]. Alcohol use disproportionately affects people of young age, as 25% of deaths among males aged 15 to 29 years are alcohol-relatable and approximately 10% of the deaths among females in this age group [2]. Young people (15-24 years) show a higher prevalence of heavy episodic drinking (HED) than among the total population and HED peaks at student age (20–24 years) [3]. In this regard, it is known that university and college students drink more than their non-student peers [4–6]. Furthermore, students tend to be more at risk for alcohol-related problems, such as alcohol abuse and alcohol dependence [5]. Also among Belgian university students, alcohol use is common. This is illustrated by the ‘Head in the Clouds?’ (HITC) study [7], which showed that Flemish students drink 9.2 glasses on average every week and that 25% of the students binge drink at least once a month (which correlates to 6 alcoholic beverages in 2 h for males and 4 alcoholic beverages in 2 h for females [8, 9]).

Students often tend to overestimate their peers' alcohol use, a phenomenon known as misperception [10–12]. This tendency is driven by students' sensitivity to social norms and their perceptions of their environment. These misperceptions can lead to even higher levels of alcohol consumption [11, 12]. The theory that behavioral norms influence behavior is called social norms theory, initially described by Perkins and Berkowitz in the context of alcohol use among students four decades ago [13] but subsequently studied in various medical and social contexts [14]. Understanding this concept has led to the development of the social norms approach (SNA), a widely used prevention strategy aimed at promoting positive behavioral change [15]. In SNA interventions, misperceptions are corrected by exposing individuals to the actual behavior observed in their peers, known as the descriptive norm. This exposure can lead to changes in individual behavior aligned with the descriptive norm [13, 16]. For an SNA intervention to have a positive impact, there must be negative health behavior in the target group, an overestimation of this behavior among peers, and a connection between this behavior and the misperceptions [17].

Multiple studies have shown the positive effect of SNA interventions on the alcohol use of students. In both the context of the US [10, 11] and Europe [12, 13], these types of interventions have led to a reduction in misperceptions and/or a decrease in alcohol consumption. However, many studies which apply SNA, lack a pre-intervention measurement for the development of credible social norm messages and do not include the participation of the target group in the intervention development [14], which is specifically important to develop an SNA intervention that is credible and appealing to the target group, to generate a higher impact. Furthermore, as also set out in a ‘critical appraisal of the SNA’ by Dempsey et al. [14], many SNA studies do not include a control group and/or a post-intervention survey, which is of great importance for a thorough effect assessment. Dempsey and his colleagues also emphasise the inclusion of a process evaluation, for improving the implementation process of the intervention. As a consequence, many SNA studies cannot sufficiently analyse and evaluate SNA interventions.

In Flanders, the Dutch part of Belgium, students also have misperceptions regarding the alcohol use of their peers. The ‘Head in the Clouds?’ study of 2021 shows that almost 52% of Flemish students think that the average student is drunk at least once a week, whereas, in reality, this is only the case in 11% of the students [7]. Another study showed that whereas almost 55% of the female students indicated not having been drunk in the last two months, only 4.2% thought this was true [12]. Furthermore, male students reported not having drunk more than seven alcoholic beverages on one occasion in the past two weeks, while fellow students thought they drank at least fourteen drinks [12].

Considering this overestimation of the descriptive norm regarding alcohol use among Flemish students, and the positive effects of SNA interventions demonstrated by earlier research, the implementation of an SNA intervention among Flemish students appears to be appropriate. Therefore, this study aimed to develop, implement and evaluate an SNA intervention for Flemish students, more specifically, University of Antwerp students, to correct their misperceptions regarding the alcohol use of their fellow students and subsequently, decrease their alcohol consumption.

## Methods

To enhance the quality and implementation process of the study, the above-mentioned pitfalls regarding SNA studies were considered. Therefore, a baseline measurement and participation of the target group were incorporated, to make sure the intervention met the expectations of the target group. In addition, we made use of a control group, post-intervention measurement and thorough process evaluation, so that the outcome and process assessment were fostered. The article starts with a description of the development and implementation of the SNA intervention in this method section. Subsequently, the results of the process evaluation and outcome assessment will be presented in the results section of the article.

In Flanders, three main university cities can be distinguished, with a distance of around 50 kms between them. Within this study, we focused on two of these cities: Antwerp and Ghent, in which University of Antwerp students formed the intervention group and Ghent University students the control group. The University of Antwerp has around 24,000 students, whereas the University of Ghent has around 51,000 students. In collaboration with the University of Antwerp, the city of Antwerp and the University of Ghent, an SNA intervention in the form of a social media campaign has been developed and evaluated within this study.

### Intervention

#### Social norm messages

The SNA intervention was developed as a campaign based on social norm messages and was spread among University of Antwerp students through social media and reusable cups for student activities. For the development of accurate social norm messages regarding alcohol use, data from the ‘Head in the Clouds’ study (HITC) measurement of 2021 were used. HITC is a four-yearly inter-university survey regarding substance use in Flanders [7]. To formulate social norm messages, answers of the University of Antwerp students on categorical questions regarding alcohol consumption and perception of alcohol consumption of peers of this HITC survey were used. Specifically, the questions addressed how often students consumed more than 6 glasses (males) or 4 glasses (females) in 2 h in the past 12 months, how often they drank enough alcohol to feel drunk in the last 12 months, how often they drank alcohol in the last 12 months (first question of the AUDIT-C, the short version of the Alcohol Use Disorder Identification Test, developed by the World Health Organisation [15]), the amount of glasses they drank on a typical day (second question of the AUDIT-C), the frequency of drinking spirits in the last 12 months and the statement ‘I have a problem with ordering non-alcoholic drinks at student activities’, with a 5-point Likert scale as answer option. In addition, the results of categorical questions regarding students’ perception of alcohol consumption of peers were used to assess if a discrepancy between the descriptive norm and perceived norm existed. These questions were: how often students think that the average student drank alcohol in the last 12 months, how often students think that the average student drank more than 6 glasses (males) or 4 glasses (females) in 2 h in the last 12 months and how often students think that the average student drank enough alcohol to feel drunk in the last 12 months.

In total, 2963 University of Antwerp students took part in this survey, of which 1866 remained after the exclusion of respondents older than 25 years (*n* = 507) and students who did not complete the questions regarding social norms and alcohol (*n* = 590). Of these students, 78.6% drank alcohol at most once a week in the last 12 months. However, this was underestimated, since only 39.2% of the respondents thought this was true and  60.8% of the respondents had the perception that their peers drank alcohol twice a week or more frequently. These results show the discrepancy between the descriptive norm and the perceived norm. Similar results were seen in questions regarding binge drinking and drunkenness.

Subsequently, the following social norm messages were formulated based on the results of the baseline measurement (translated from Dutch):

- ‘Binge drinking is unpopular among most Antwerp students’
- ‘64.3% of Antwerp students get drunk maximum once a month only’
- ‘78.6% of Antwerp students drink alcohol maximum once a week’
- ‘65.1% of Antwerp students only drink 1 or 2 drinks on one occasion’
- ‘72.9% of Antwerp students have no problem ordering non-alcoholic drinks at student activities’
- ‘78.9% of Antwerp students drink spirits maximum once a month only’

These social norm messages were formulated following the SNA guidelines of the National Social Norms Center of Michigan State University (US) [16, 17], which state that the normative messages should be formulated in a positive, inclusive and empowering way and should focus on stimulating positive behaviour. Subsequently, these messages were tested with student representatives of the University of Antwerp. We made use of several student boards, amongst which the umbrella student association for the campuses outside of the city center, the umbrella student association for the city center campus, the students responsible for the official student magazine, the student council, and the general student meeting, which were all involved in the process. They were also presented the Social Norms messages and the different concepts of the campaign for their feedback, which we included in the intervention.

## Campaign development

A campaign was developed based on the above social norm messages. The communication department of the City of Antwerp, in collaboration with a design agency named Shtick, and closely supported by stakeholders including the communication service of the University of Antwerp, the study advice and student counseling service of the University of Antwerp, the researchers of the study team, and student representatives, contributed to its development.

Various prototypes for the social media campaign were developed, which were presented to the above stakeholders during the development process. During these discussions, the choice was made to use humorous memes that matched the social norm messages. Also in the further development of the campaign, the opinion of the stakeholders was taken into account, mostly in the form of consultations (level 4 of the participation ladder of Arnstein [18]). Apart from the use of social norms to correct misperceptions in the intervention group, other behavioural change techniques were used, such as increasing knowledge and raising awareness of alcohol use.

**Fig. 1:**
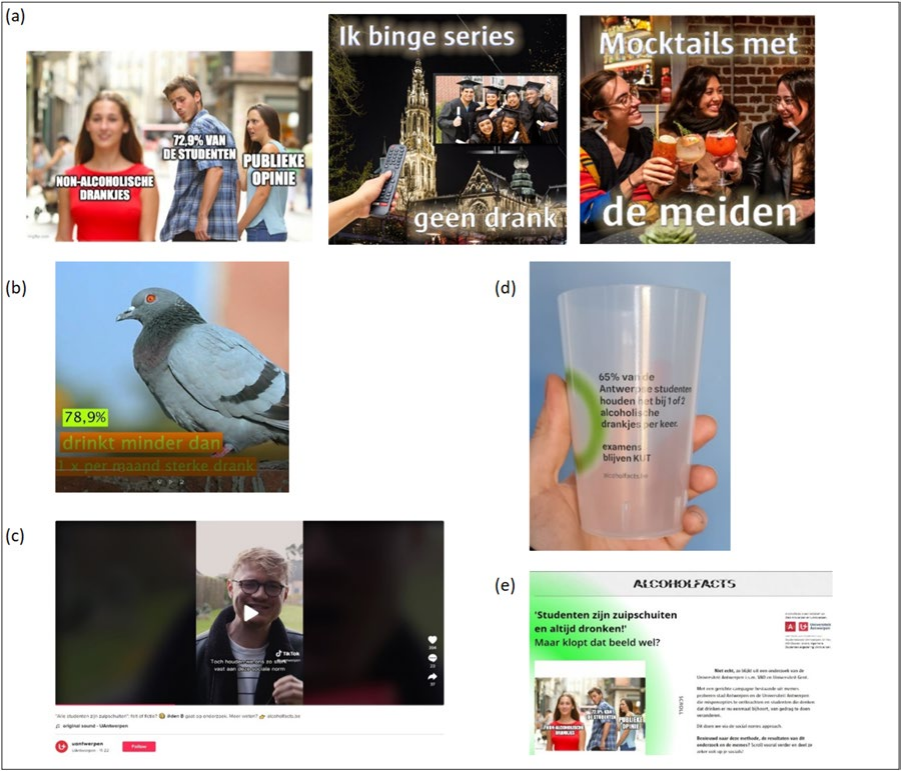
Examples of the campaign components of a social norms approach intervention on alcohol use among Flemish university students in 2022-2023

The final campaign, named ‘Alcoholfacts’, consisted of seven static memes (Fig. 1a), two video memes (Fig. 1b), a vlog by an Antwerp student (Fig. 1c) and reusable cups printed with social norm messages for use at student activities (10,000 cups, Fig. 1d). Furthermore, a website was developed (www.alcoholfacts.be, Fig. 1e) where students could find more information about the campaign, alcohol use in general, contact details of help organisations and download materials for the spreading of the campaign via their own social media channels [19]. The social norm messages were incorporated into the campaign by linking every social media post to one of the SNA messages in the caption. Furthermore, every social media post also included a reference to the campaign website.

The campaign was launched on 16 November 2022 and initially ran for four weeks. In this period, the City of Antwerp advertised the (video) memes on their social media channels, via TikTok and Instagram. On TikTok, the videos were both posted as organic and paid content (500 euros budget). The latter was targeted at Flemish 18–24 years old with an interest in travel, sports & outdoor, food & beverage, beauty, news & entertainment, higher education or games. In addition, the (video) memes were spread via paid advertisements on Instagram (Reels, Stories, Feed and Explore). A budget of 800 euros was spent to increase the reach of the campaign and 300 euros were spent to raise the number of videos which were played to completion. The advertisements were targeted at people 18–25 years old from Antwerp and its surroundings (40 kms), with an interest in university/college. Furthermore, the student vlog was posted on the TikTok (organic) and Instagram channels (paid) of the University of Antwerp. For Instagram, a budget of 200 euros was spent on clicks to the website, with students aged 18–25 years from Antwerp and its surroundings (40 kms, the Netherlands excluded) and an interest in university/college as the target group. Moreover, student associations of the University of Antwerp were asked to spread the campaign on their social media channels. In addition, the 10,000 reusable cups were distributed among student associations in this period and subsequently used in their student activities. Finally, a combined press release from the city of Antwerp and the University of Antwerp was published, which was picked up by media (radio and newspapers).

During the running time of the campaign, it was decided to run the campaign for a second time, to increase the chance of exposure. Therefore, a boost campaign was launched on 20 March 2023, with a running time of two weeks. During this boost campaign, the city of Antwerp posted advertisements of the memes on their Instagram channel, with a budget of 1185 euros and reach as objective. The same targets were used, except for a smaller range of 20 kms around Antwerp instead of 40 kms. The student vlog was posted on the Instagram channel of the University of Antwerp with a budget of 200 euros and clicks to the website as the objective. Here, the same group as the initial campaign was targeted.

### Study design and population

A quasi-experimental study design was used to assess the effect of the developed and implemented SNA intervention on the alcohol use and misperceptions about the alcohol use of peers of Flemish students. The allocation of the SNA intervention was non-randomised: students of the University of Antwerp formed the target group of the social media campaign, whereas Ghent University students served as the control group.

### Measurements

#### Data collection

Anonymised data from the HITC survey edition 2021 [7] of the students of the University of Antwerp and Ghent University were used to assess characteristics at baseline (before the campaign). This survey includes questions on socio-demographics, alcohol use, other substance use, perception of alcohol and substance use, and mental well-being. In addition, an anonymous post-intervention survey was developed. In this post-intervention survey, similar questions regarding alcohol use and perception of alcohol use as in the baseline measurement were used, however, adopted to the appropriate period: these questions were not asked over the last 12 months as in the baseline measurement, but over the last 5 months, to cover the period since the release date of the SNA campaign. Furthermore, questions regarding socio-demographics, other substance use and mental well-being, were also incorporated in the post-intervention survey and asked in the same way as in the baseline measurement. Finally, the post-intervention survey also covered questions regarding the process evaluation. The survey consisted of two different versions, one for the University of Antwerp students and one for the Ghent University students, due to minor differences in reply options of socio-demographic questions. The survey was created using Qualtrics software, version 2022 [20]. Subsequently, the survey was conducted among students of both universities after the running time of the campaign, between 17 April 2023 and 11 May 2023. Recruitment of students took place via email and student portals, similar to the recruitment process of the HITC survey in 2021.

Students of the University of Antwerp and Ghent University of 17–25 years old who filled in the baseline and/or endline (post-intervention) survey were included in the study. Ghent University students who were exposed to the campaign, as indicated by the post-intervention survey, were excluded from the study. Students from the intervention group who were not exposed to the campaign, or had unknown exposure to the campaign, were still included in the outcome analyses, as is common in quasi-experimental studies [21]. By including all targeted students in the intervention group, students who were exposed subconsciously to the campaign were also included in the analyses. Moreover, this approach allowed to test the potential effects of the campaign policy, rather than the potential effects of the intervention on an individual level, which is often more relevant for policymakers and researchers. This approach therefore increased the external validity.

#### Outcome variables

A process evaluation was performed according to the Medical Research Council guidance for process evaluation of complex interventions [22, 23]. In this study, three different key components of process evaluations were analysed: the implementation of the intervention, the mechanisms of impact (level of satisfaction, relevance, and perceived benefit), and the context. Data for the process evaluation were obtained by asking various questions regarding the opinions and experiences of the students concerning the campaign in the post-intervention survey. For example, the level of satisfaction of the campaign as a whole and the different components of the campaign were asked by using a scale from 0 (low level of satisfaction) to 10 (high level of satisfaction). Also, statements regarding the credibility, attractiveness, relevance and perceived benefit were suggested to the students, with a 5-point Likert scale as answer option. For example, the following statements were presented: ‘I found the campaign credible’, and ‘My perception of the alcohol consumption of my peers decreased due to the campaign’. Furthermore, the process evaluation was complemented by data from the communication departments of the City of Antwerp and the University of Antwerp regarding the spent budget, reach (number of persons reached), impressions (number of impressions of the advertisements) and clicks to the website. These metrics were obtained through Google Analytics and Meta Insights.

The primary outcome assessed in the study was a change in students' alcohol consumption, measured by the number of glasses consumed per week during course periods. This continuous variable was computed in the same way as in the HITC study [7], by combining the answers to two specific questions from both the baseline and endline measurement, namely: how often students drank specific types of alcoholic beverages (light beers, strong beers, wine, spirits) during course periods in the last 12 months (baseline) or last 5 months (endline) (never, monthly or less, once a week, 2–3 times a week, 4 times a week or more) and the number of glasses of these specific beverage types per day during the questioned periods (1–2 glasses, 3–4 glasses, 5–6 glasses, 7–9 glasses, 10 or more glasses). First, the frequency question was quantified in days of drinking over the questioned period (12 months for baseline measurement and 5 months for post-intervention measurement). Second, the number of glasses per drinking day was quantified using the same method as Wicki et al., 2006 [24]. Here, the answers for heavy beers were multiplied by two to correct for the higher percentage of alcohol and higher volume in comparison with other beverages. Third, the total number of drinking days and the number of alcoholic drinks per day were multiplied to calculate the total amount of alcohol consumed during course periods. Next, an outlier correction based on adjusted boxplot analyses [25] using RStudio version 2023.06.0 [26] was performed. The calculated total was then divided by 24 (baseline measurement) or 11 (post-intervention measurement) to come to the number of glasses of alcoholic beverages per week in course periods, as the course period was set at 24 weeks for the baseline measurement (the questions were asked over the academic year which includes 24 weeks of courses) and at 11 weeks for the post-intervention measurement (here, the questions were asked over 5 months, December 2022 till May 2023, which included 11 weeks of courses).

The secondary outcome measured was a change in students' perception of their peers' alcohol consumption, quantified by the number of glasses consumed per week. This was questioned with the following open question: ‘How many alcohol glasses do you think an ordinary student drinks on average in one week during course periods?’

#### Covariates

Potential confounders were selected based on results of the baseline measurement, literature review, expert opinions and directed acyclic graphs (DAGs) that were drawn a priory to identify covariates that could influence the effect of the intervention (see Additional file 1). The following variables were obtained from both the baseline and post-intervention survey and analysed to assess confounding effects: sex (men/woman), age (open question), faculty (the specific faculties of the University of Antwerp and Ghent University, respectively, which were then recoded to be able to combine), type of education (bachelor program, master program, bridging program or other), living situation during weekdays (at parents or independently), working status (working less than 20 h per week, working more than 20 h per week, not working, which was subsequently recoded into working or not working), religion (Christian, Jewish, Islamic, Hindu, Buddhistic, no religion, other, which was subsequently recoded into Christian, Islamic, other and no religion), importance of religion (5-point Likert scale on importance, which was recoded into unimportant and neutral to important), being a fraternity member (never, in the past, passive member at the moment, active member at the moment, organising member at the moment, which was subsequently recoded into being an active/organising member at the moment or not), other substance use (see below for specific variables), mental wellbeing (see below for specific variables) and exposure to other campaigns (yes, no). When significant differences in these potentially confounding variables existed between the intervention and control group, they were added to the analyses to correct for a potential confounding effect in the outcome assessment.

Regarding other substance use as potential confounder, students were asked if they used tobacco, tranquillizers (non medical use), cannabis or stimulating medication (non medical use) in the past 12 months (yes, no). Furthermore, ever use of other illegal drugs than cannabis was also questioned (yes, no).

To assess the potential covariate mental wellbeing, life satisfaction was questioned using the Cantril ladder, with 0 representing the worst possible life and 10 presenting the best possible life [27]. In addition, psychological distress was surveyed by the Kessler-6 psychological distress scale (Cronbach α = 0.88). This scale is a 5-point Likert scale, ranging from 0 (never) to 4 (very often), with a total score range of 0–24 and a higher score meaning more psychological distress [28].

#### Sample size

Due to the small effect sizes of SNA interventions on alcohol consumption in alcoholic beverages per week (our primary outcome) in previous research [29, 30], a small effect size of 0.15 was assumed. Taking into account a significance level of 0.05 (two-sided test), and a power of at least 80%, a minimum of 699 subjects in the intervention group and 699 subjects in the control group were required to participate in the study, based on a t-test for comparing two independent means, calculated with G*Power 3.1.9.4 software.

#### Analytic strategy

Descriptive statistics were used to analyse baseline and endline characteristics. They were expressed as proportions for categorical variables and as medians [interquartile range, IQR] for continuous variables, due to the non-normal distribution of the continuous variables. To analyse differences in characteristics between the intervention and control group, Pearson’s chi-squared tests were used for categorical variables and Mann–Whitney U tests for continuous variables. Furthermore, to examine the impact of the intervention on the primary and secondary outcome variables, a Difference-in-Difference (DiD) approach was used. DiD is a common model for quasi-experimental study designs, when randomised controlled trials are not feasible [31]. It compares the change in outcome in an intervention group before and after exposure to the intervention, while accounting for a concurrent change in the control group not receiving the intervention [31, 32]. In our study, the DiD model was integrated into a multiple linear regression model (following an intention-to-treat analysis, ITT), one for analysing the effect of the intervention on alcohol consumption (primary outcome) and one for the perception of alcohol consumption of peers (secondary outcome). In addition, variables that significantly differed between the intervention and control group at baseline or endline were analysed for the univariable association with the primary and secondary outcome, respectively. These univariable analyses were performed using Mann–Whitney U tests for variables with two categories and Kruskal–Wallis tests for variables with more than two categories. Statistically significant variables from the univariable analyses were included in the multiple linear regression models. The models were built using backward elimination. Subsequently, these multiple linear regression models were bootstrapped, because the assumptions of normality and homoscedasticity of residuals were not met. In addition, an exploratory subgroup analysis for sex was performed, due to the hypothesis that SNA interventions can have a different impact on females compared to males [33]. Also, a subgroup analysis on students from the intervention group who were exposed to the campaign (a per-protocol analysis, PP), was performed, to test for a difference in intervention effect between exposed and non-exposed students. The models of the subgroup analyses were built in the same way as the models of the main analyses.

The significance level for all statistical tests was set at α = 0.05. Statistical analyses were performed using IBM SPSS Statistics for Windows, Version 29.0.

## Results

In this section we will firstly discuss the results of the process evaluation of the campaign. Next, the study participants and outcome assessment will be presented. Finally, the results of the subgroup analyses will be addressed.

### Process evaluation

#### Implementation

**Fig. 2:**
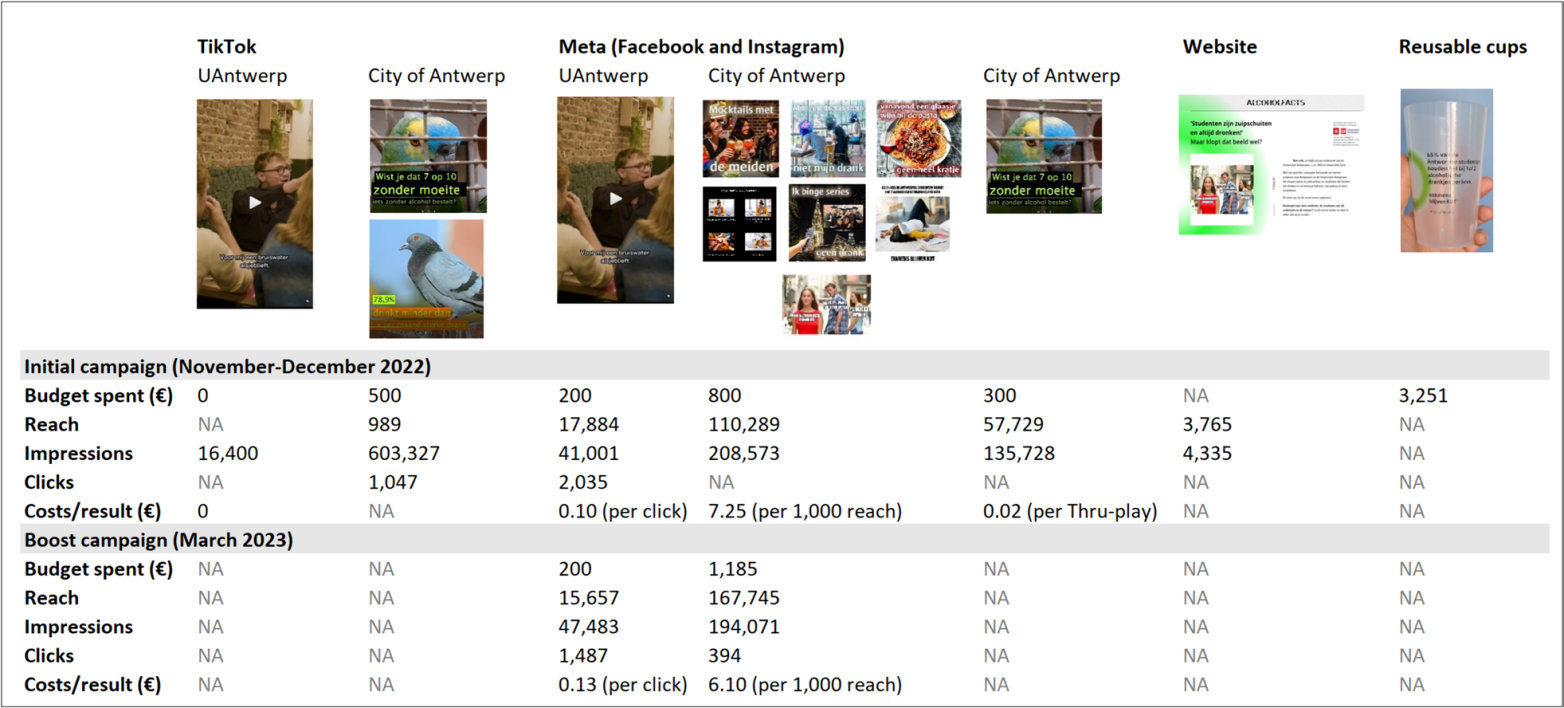
Detailed metrics of the different components of a social norms approach intervention on alcohol use among Flemish university students in 2022-2023

During the initial campaign, more than one million impressions of the different components of the campaign were achieved on TikTok and Meta (Facebook and Instagram), with more than 3,000 clicks to the website as a result. In addition, the boost campaign resulted in 242,554 impressions on Meta in total and 1881 clicks to the website. During the initial campaign, the website reached 4,335 impressions among 3,765 individuals. Unfortunately, details regarding website views during the boost campaign are lacking*.* Figure 2 shows a more detailed overview of the metrics of the various components of the campaign, including the costs/result. Moreover, 10,000 reusable cups were distributed to the student associations of the University of Antwerp during the initial campaign and have since been used continuously at various student events. Furthermore, the campaign was also spread by student associations  on their social media channels, however, the metrics of these organic posts were not available for analysis.

Analysis of the post-intervention survey showed that 36.3% (556/1530) of the University of Antwerp students who filled in the questionnaire, were exposed to at least one component of the campaign. Here, female students were exposed more frequently, as 38.1% of the female students (350/981) and 33.7% of the male students (206/611) reported they had seen (part of) the campaign. Of the exposed students, 48.4% were exposed via Instagram, 41.0% via Facebook, 15.5% via TikTok, 4.5% via television or radio and 16.7% via other channels, such as events, student portals and the reusable cups at student events. Furthermore, more than a quarter of the students (28.8%) who were exposed indicated they had seen the campaign via multiple channels.

The static memes (Fig. 1a) were the most seen component of the campaign, with 72.3% (402/556) of the exposed students indicating they had seen at least one of the memes. This was followed by the reusable cups (Fig. 1d, seen by 27.9%) and the student vlog (Fig. 1c, seen by 24.8%). Only 7.6% were exposed to the video memes (Fig. 1b), 6.8% to the website (Fig. 1e) and 8.6% reported they had heard about the campaign via television or radio. In addition, most students (63.8%) were exposed to only one campaign component.

### Mechanisms of impact

#### Level of satisfaction

Almost 75% of the students who were exposed to the campaign, found the campaign credible (387/525). However, most of the students (54.8%, 238/527), did not find the campaign appealing. In addition, the exposed students rated the overall campaign with a median score of 6 out of 10 [IQR 2.0]. Analysing student satisfaction with the different components of the campaign, showed that the student vlog and the reusable cups were rated the highest (median score of 7.0 [IQR 2.0]) and 7.0 [IQR 3.0], respectively) and the campaign website, memes and video memes were rated lower (median score of 7.0 [IQR 1.3], 6.0 [IQR 3.0] and 6.0 [IQR 2.0], respectively).

#### Relevance of the campaign

The majority of the exposed students agreed with the relevance of the campaign (90%, 480/528), and only a quarter of the students reported that too much attention is given to the subject of alcohol consumption in their opinion.

#### Perceived benefits of the campaign

Almost 58% (301/524) of the exposed students indicated that their perception of the alcohol consumption of their peers decreased (closer to the descriptive norm) due to the campaign. However, only 11.6% (61/526) reported to have reduced their own alcohol consumption due to the campaign. Noteworthy here is that the student vlog and website had the biggest impact on the self-reported decrease of the perceived norm (76.5% or 26/34 of the students and 70.8% or 92/130 of the students, respectively), whereas the reusable cups had the lowest impact (52.7% or 68/146 of the students). Furthermore, the campaign has made 61.2% (191/502) of the students reflect on the alcohol consumption of their peers and 38% (191/502) on their own alcohol consumption. Only in a small proportion of the exposed students, the campaign had a negative health outcome: 14% (74/527) of the exposed students indicated that the campaign has made them aware that peers drank more alcohol than they previously thought and in only 2.1% (11/528) of the students, the campaign has encouraged students to drink more alcohol.

#### Context

There are multiple ways in which the context potentially affected the implementation of the campaign. For instance, the initially developed campaign materials were not suitable for spreading via the University of Antwerp channels, due to not fitting the communication style guidelines of the University. Therefore, the University of Antwerp's social media channels could not be used as planned. However, the student vlog was suitable and therefore spread via the University of Antwerp channels. Furthermore, a potential effect of exposure to other campaigns targeting alcohol use during the study period should be taken into account. However, only 3.3% (41/1261) of the University of Antwerp and 4.8% (87/1830) of the Ghent University students reported they were exposed to other campaigns on alcohol use during the study period.

### Study participants

#### Participant flow

**Fig. 3:**
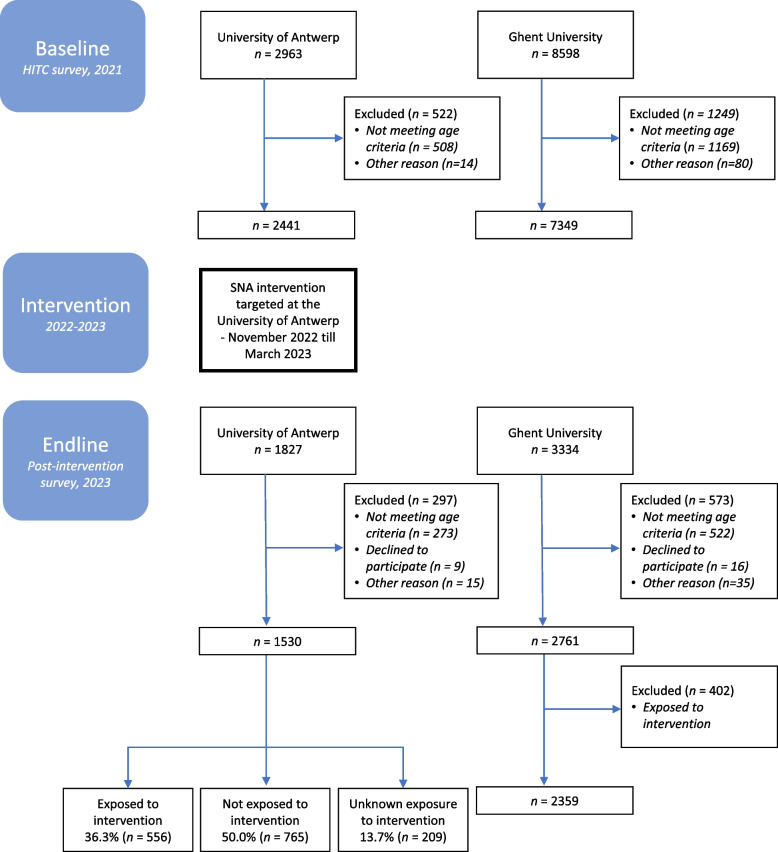
Flow diagram of participants of a social norms approach intervention on alcohol use among Flemish university students in 2022-2023

Figure 3 shows the participation flow of the study. At baseline, there were 2,963 respondents from the University of Antwerp and 8,598 from Ghent University. After applying the exclusion criteria (age above 25 years and outliers for the primary outcome based on adjusted boxplot outlier correction), 2,441 students of the University of Antwerp and 7,349 of Ghent University were included. At endline (post-intervention), 1,827 University of Antwerp students filled in the questionnaire, which corresponds to a response rate of 7.63% (1,827/23,944 total students). At Ghent University, a response rate of 6.5% was obtained (3,334/51,237 total students). Here, students above 25 years old were also excluded, as well as students who did not fill in the questionnaire seriously (2 participants of the post-intervention survey from the University of Antwerp, based on comments on open questions which said they were not taking the survey seriously). Furthermore, based on the adjusted boxplot outlier correction for the primary outcome, 13 University of Antwerp students and 35 Ghent University students were excluded from the post-intervention survey. Moreover, the 402 students (14.6%) of the University of Ghent who were unintentionally exposed to the intervention, were also excluded from the study. Therefore, at endline, 1,530 University of Antwerp students and 2,359 Ghent students were included in the study.

#### Characteristics of study participants

Table 1 presents participant characteristics, both as baseline and endline. All potential confounders are included in the table. Both in the intervention and control group, more females participated, with 61.1% and 62.0% females, respectively, at baseline, and 60.0% and 60.6%, respectively, at endline. At baseline, the median age was 21.0 [IQR 3.0] for both the intervention and control group. However, at endline, the median age of the University of Antwerp students was significantly lower than that of Ghent University students, namely 20.0 [IQR 3.0] compared to 21.0 [IQR 3.0]. Both at baseline and endline, significant differences were found between the intervention and control group for the distribution of faculty, type of education, living situation during weekdays, religion and last-year cannabis use. In addition, at endline, there was a significant difference between the intervention and control group in the distribution of the importance of religion, being an active fraternity member and exposure to other alcohol-related campaigns, next to age.

**Table 1:** Characteristics of Flemish university students before (baseline, 2021) and after (endline, 2023) a social norms approach intervention on alcohol use

**Characteristic**	**Baseline**		**Endline**	
	**Intervention Group**	**Controlgroup**	**Intervention Group**	**Controlgroup**
	*n* = 2441	*n* = 7349	*n* = 1530	*n* = 2359
**Sex, n(%)**				
Male	949 (38.9)	2789 (38.0)	611 (40)	929 (39.4)
**Age (years)**				
Median [IQR]	21.0 [3.0]	21.0 [3.0]	20.0 [3.0]	21.0 [3.0]
**Faculty, n(%)**				
Medicine and Health Sciences	377 (15.5)	1293 (17.6)	247 (16.2)	410 (17.4)
Veterinary and Pharmaceutical Sciences	361 (14.8)	494 (6.7)	239 (15.6)	154 (6.5)
Engineering Sciences	380 (15.6)	1450 (19.8)	228 (14.9)	413 (17.5)
Exact Sciences	270 (11.1)	518 (7.1)	210 (13.7)	206 (8.7)
Economics	304 (12.5)	893 (12.2)	142 (9.3)	230 (9.8)
Political, Social and Educational Sciences and Psychology	226 (9.3)	1350 (18.4)	113 (7.4)	451 (19.1)
Linguistics and Philosophy	297 (12.2)	794 (10.8)	218 (14.3)	335 (14.2)
Law and Criminology	224 (9.2)	538 (7.3)	131 (8.6)	159 (6.7)
**Type of education, n(%)**				
Bachelor program	1548 (63.4)	4106 (55.9)	1064 (69.6)	1403 (59.5)
Master program	752 (30.8)	2782 (37.9)	376 (24.6)	809 (34.3)
Bridging program	126 (5.2)	404 (5.5)	82 (5.4)	116 (4.9)
Other	15 (0.6)	57 (0.8)	7 (0.5)	29 (1.2)
**Living situation weekdays, n(%)**				
Parental home	1567 (64.2)	2962 (40.3)	922 (61.2)	845 (36.6)
Independently	872 (35.8)	4385 (59.7)	585 (38.8)	1466 (63.4)
**Working status**				
Working	721 (29.7)	1709 (23.5)	607 (39.7)	772 (32.8)
**Religion, n(%)**				
Christian	779 (32.0)	2740 (37.3)	367 (24.3)	704 (30.4)
Islamic	94 (3.9)	143 (1.9)	59 (3.9)	33 (1.4)
No religion	1449 (59.4)	4184 (57.0)	1017 (67.3)	1485 (64.2)
Other	116 (4.8)	275 (3.7)	69 (4.6)	91 (3.9)
**Importance of relgion**				
Neutral to important	514 (21.1)	1378 (18.8)	216 (44.3)	291 (36.0)
**Active fraternity member, n (%)**				
Yes	274 (14.7)	812 (12.5)	305 (20.5)	360 (15.9)
**Last-year-Tobacco use, n(%)**				
Yes	580 (24.0)	1767 (24.2)	356 (31.5)	464 (29.6)
**Last-year-NMUPS, n(%)**				
Yes	90 (4.6)	264 (4.0)	76 (6.0)	85 (4.7)
**Last-year Nonmedical Tranquilizer use, n(%)**				
Yes	63 (3.2)	170 (2.6)	86 (7.6)	106 (6.8)
**Last-year-Cannabis use, n(%)**				
Yes	448 (23.2)	1768 (26.7)	285 (25.2)	411 (26.3)
**Ever-use illegal drugs, other than cannabis, n(%)**				
Yes	269 (14.0)	808 (12.2)	172 (15.2)	233 (14.9)
**Life satisfaction (Cantril scale)**				
Median [IQR]	6.0 [2.0]	6.0 [2.0]	7.0 [2.0]	7.0 [2.0]
**Psychological distress (Kessler-6 scale)**				
Median [IQR]	11.0 [8.0]	11.0 [8.0]	9.0 [7.0]	9.0 [7.0]
**Exposure to other alcohol campaigns**				
Yes	NA	NA	41 (3.3)	87 (4.8)
**Participation in Tournée Minérale (no alcohol for 1 month)**				
Yes	NA	NA	186 (14.8)	304 (16.6)

We also examined the number of students in the target group who overestimated the weekly alcohol consumption of their peers, measured in median glasses per week. Within the intervention group at baseline, an overestimation of the actual norm of alcohol use was seen in 69.3% (1691/2441) of the students. Therefore, we assumed that more than two-thirds of the intervention group at baseline had misperceptions about the alcohol consumption of their peers, which made them sensitive to the campaign due to targeting the correction of these misperceptions. Here, a slightly higher percentage of overestimation was seen in female students (71.1%, 1058/1489) than in male students (66.7%, 633/949).

### Outcome evaluation

#### Intervention effect on alcohol consumption

**Fig. 4:**
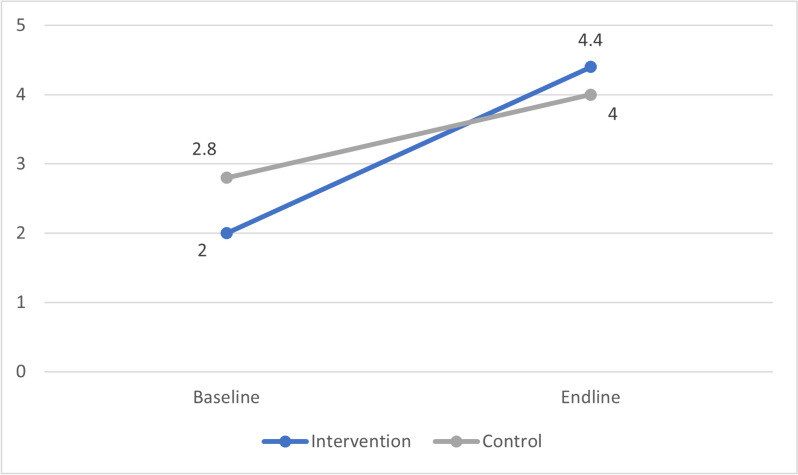
Alcohol consumption in median glasses per week during course periods among Flemish university students before (2021) and after (2023) a social norms approach intervention on alcohol use

Analysing the primary outcome of alcohol consumption in glasses per week in course periods showed a median of 2.0 [IQR 7.1] glasses per week for students of the University of Antwerp and 2.8 [IQR 7.8] for the Ghent University at baseline. At endline, the alcohol consumption increased to 4.4 [IQR 11.9] glasses per week for the University of Antwerp students and 4.0 [IQR 12.3] for Ghent University students, as shown in Fig. 4.

Table A2.1 shows the results of the univariate analyses between the variables with significant differences between the intervention and control group at baseline and/or endline including the variable sex, and the primary outcome (see Additional file 2). Table 2 presents the results of the subsequent bootstrapped multiple regression analysis. The model was statistically significant with F(10,7347) = 144.273, *p* < 0.001 and R^2^ = 0.164 and indicated that time significantly predicted a higher alcohol consumption, however, the intervention (bootstrapped *p* = 0.541; B = -0.212, bootstrapped CI = -0.433 to 0.859) and interaction between time and intervention (DiD) did not predict the outcome variable (bootstrapped *p* = 0.741; B = -0.32, bootstrapped CI = -2.101 to 1.534). This result indicates that the intervention did not contribute to a change in alcohol consumption post-intervention. In other words, the influence of time was greater than the influence of the intervention. This finding could be explained by the effects of the lockdown period in 2021 due to the COVID-19 pandemic, because the baseline measurement showed that 67% of the students of the University of Antwerp and Ghent University reported lower alcohol consumption during the COVID-19 pandemic [7].

**Table 2:** Results of the bootstrapped multiple regression analysis of a social norms approach intervention (2023) on alcohol use among Flemish university students

Independent variable	B Unstandardized Coefficient	β Standardized Coefficient	Bootstrapped *p*-value	Bootstrapped 95% Confidence Interval
Group (Intervention)	0.212	0.007	0.541	[-0.433, 0.859]
Time (Endline)	1.217	0.030	0.044*	[0.031, 2.419]
Group * Time (Intervention * Endline)	-0.320	-0.005	0.741	[-2.101, 1.534]
Sex (Male)	6.071	0.236	< .001*	[5.453, 6.723]
Religion	-0.839	-0.700	< .001*	[-1.120, -0.549]
Importance of religion (Neutral to important)	-1.488	-0.490	< .001*	[-2.118, -0.836]
Fraternity member (Active)	1.777	0.050	0.002*	[0.896–2.711]
Living situation weekdays (Independently)	3.022	0.120	< .001*	[2.525–3.547]
Working status (Working)	1.594	0.056	< .001*	[0.950–2.204]
Last-year use of cannabis (Yes)	6.710	0.243	< .001*	[6.010–7.461]

^*^Statistically significant

## Intervention effect on the perceived norm of alcohol consumption

**Fig. 5:**
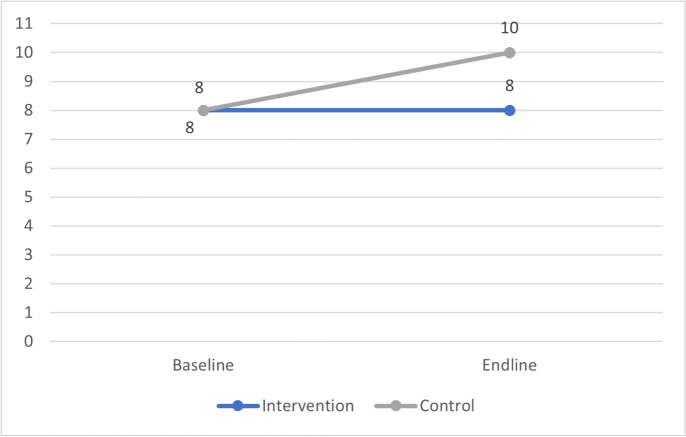
Perceived norm of alcohol consumption in median glasses per week during course periods among Flemish university students before (2021) and after (2023) a social norms approach intervention on alcohol use

When evaluating the perception of alcohol consumption of other students, the secondary outcome of the study, an increase in perceived norm stands out for the control group: whereas students of Ghent University had the perception that an ordinary student drinks a median of 8.0 [IQR 9.0] glasses per week on average at baseline, this increased to 10.0 [IQR 9.0] glasses per week at endline. On the other hand, the perceived norm of the University of Antwerp remained equal over time (from 8.0 [IQR 7.0] median glasses at baseline to 8.0 [IQR 8.0] at endline), as shown by Fig. 5.

Table A2.2 shows the univariate analyses of the relation between the perceived norm of alcohol consumption and the variables with a significant difference in distribution between the intervention and control group at baseline and/or endline (see Additional file 2). Also here, the variable sex was added to the univariate analysis. The subsequent bootstrapped multiple regression model significantly predicted the outcome of the perceived norm of alcohol consumption, with R^2^ = 0.009, F(8,10891) = 12.434 and *p* < 0.001, as shown in Table 3. The outcome of the analysis indicated that students of the intervention group at endline estimated the alcohol consumption significantly lower compared to students of the control group (bootstrapped *p* = 0.013; B = -1.93, bootstrapped CI = -3.620 to -0.565) and thus, closer to the actual norm. Therefore, it can be assumed that the intervention had an impact on the perception of alcohol consumption.

**Table 3:** Results of the bootstrapped multiple regression analysis of a social norms approach intervention (2023) on perception of alcohol use among Flemish university students

Independent variable	B Unstandardized Coefficient	β Standardized Coefficient	Bootstrapped *p*-value	Bootstrapped 95% Confidence Interval
Group (Intervention)	0.285	0.006	0.697	[-0.970-, 1.880]
Time (Endline)	-0.284	-0.006	0.378	[-0.968, 0.330]
Group * Time (Intervention * Endline)	-1.930	-0.029	0.013*	[-3.620, -0.565]
Sex (Male)	1.903	0.045	< .001*	[0.997, 2.864]
Fraternity member (Active)	1.501	0.026	0.041*	[0.262, 3.121]
Living situation weekdays (Independently)	1.088	0.027	0.002*	[0.407, 1.688]
Working status (Working)	1.106	0.024	0.012*	[0.288, 1.939]
Last-year use of cannabis (Yes)	2.461	0.053	< .001*	[1.585, 3.479]

^*^Statistically significant

### Subgroup analyses

#### Subgroup analysis for sex

Analysing the primary outcome for females and males separately, showed no moderation of sex. For both subgroups, the interaction between time and intervention (DiD) did not predict the outcome variable, similar to the main analysis (females: bootstrapped *p* = 0.984; B = -0.022, bootstrapped CI = -1.670 to 1.797, males: bootstrapped *p* = 0.549; B = -1.190, bootstrapped CI = -5.001 to 2.657).

**Fig. 6:**
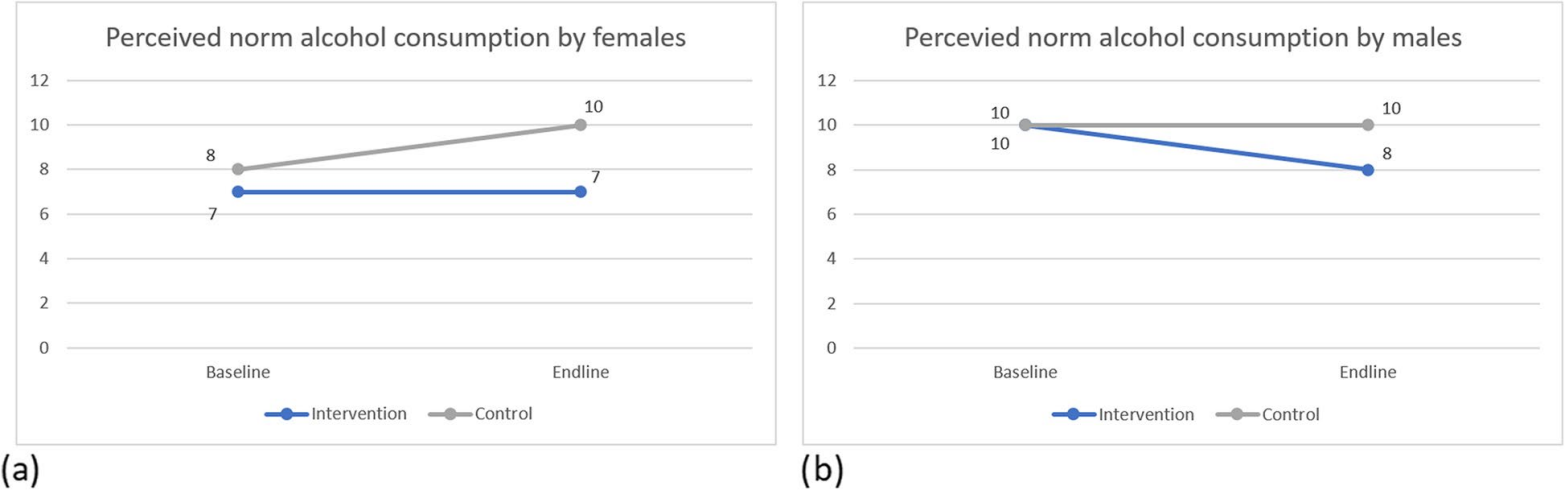
Perceived norm of alcohol consumption in median glasses per week during course periods among Flemish university students before (2021) and after (2023) a social norms approach intervention on alcohol use, separately for females (**a**) and males (**b**) . The results of the bootstrapped linear regression models on the secondary outcome for females and males separately are presented in Additional file 3. For females, the bootstrapped model on the perception of alcohol consumption (Table A3.1) was significant, with R^2^ = 0.010, F (7,6825) = 10.054, *p* < .001. According to the outcome of the model, female students from the intervention group at endline estimated the alcohol consumption of their peers significantly lower than the control group (bootstrapped *p* = 0.045; B = -1.643, bootstrapped CI = -3.297 to -0.222). However, the outcome of the significant bootstrapped linear regression model for males (R^2^ = 0.004, F (4,4110) = 3.836, *p* = 0.004, see Table A3.2), did not show a significant effect of the intervention over time (bootstrapped *p* = 0.158; B = -2.354, bootstrapped CI = -5.785 to 0.363)

In addition, an exploratory subgroup analysis of the secondary outcome for sex was performed. Figure 6 shows the change in perception for the intervention and control groups for females and males separately. Whereas the perceived norm of alcohol consumption by females remained the same in the intervention group (with a median of 7.0 [IRQ 6.0] glasses per week), it increased among females in the control group (from a median of 8.0 [IQR 7.0] to 10.0 [9.0] glasses per week), as illustrated by Fig. 6a. However, among males in the intervention group, the perceived norm decreased from 10.0 [IQR 10.0] glasses per week to 8.0 [IQR 7.0], whereas it remained the same for students from the control group (a median of 10.0 [IQR 9.0] glasses per week), as shown by Fig. 6b.

#### Subgroup analysis for exposed students

A PP subgroup analysis was performed to test for a potential intervention effect on the primary outcome among students who were exposed to the campaign. Table A3.3 shows the endline characteristics of students from the intervention group for exposed students and non-exposed students or students with unknown exposure to the campaign separately (see Additional file 3). Here, the following variables showed a significantly different distribution between exposed and non-exposed students: faculty, living situation during weekdays, being an active fraternity member, ever use of other illegal drugs than cannabis and exposure to other alcohol-related campaigns. Because ever use of other illegal drugs than cannabis was not included in the univariate analysis of the main analysis, we tested the association between ever use of other illegal drugs than cannabis and alcohol consumption, which appeared to be significant (*p* < 0.001). Therefore, this potential confounding variable was also added to the subgroup analysis. Similar to the main analysis, no significant effect of the intervention was identified according to the DiD approach among exposed students (bootstrapped *p* = 0.281; B = -1.158, bootstrapped CI = -3.221 to 0.891).

Also, a PP subgroup analysis was performed to test whether the significant intervention effect on the perception of alcohol use of peers among students who were exposed to the campaign was more pronounced than in the total intervention group. Also here, ever use of other illegal drugs than cannabis was associated with the outcome (*p* < 0.001), and therefore, this variable was also added to the subgroup analysis. Regarding the secondary outcome, no difference was observed in the perceived norm in median glasses per week between the University of Antwerp students who were exposed to the campaign (median 8.0 [IQR8.0]) and those who were not exposed or had an unknown exposure (median 8.0 [IQR8.0]). However, a bootstrapped linear regression showed a larger effect of the intervention on the perceived norm in the exposed group, compared to the overall intervention group. The model was significant, with R^2^ = 0.008, F (8,10281) = 11.454, *p* < 0.001. In the group of exposed students, the intervention led to a decrease in the perception of alcohol use of 2.15 glasses per week (bootstrapped *p* = 0.008; B = -2.147, bootstrapped CI = -3.798 to -0.538), compared to 1.93 (bootstrapped *p* = 0.013; B = -1.930, bootstrapped CI = -3.620 to -0.565) among the overall intervention group. See Additional file 3, Table A3.4, for the regression table of the PP analysis for the secondary outcome.

## Discussion

This study was the first in Belgium to develop, implement and evaluate an SNA intervention targeting alcohol consumption and the perception of alcohol consumption of peers among university students. The study aimed to conduct an evaluation of both the effectiveness and process of a social norms campaign, which was developed and implemented from a cooperation between the City of Antwerp and the University of Antwerp. The main results from the DiD analyses used in this study showed a small but significant effect of the intervention on student’s perception of the alcohol consumption of their peers. However, no significant effect on student’s alcohol consumption has been found.

Where the perception of alcohol consumption of peers remained equal in the intervention group before and after the intervention, it increased in the control group. Here, the results of the analysis indicated a significant effect of the SNA intervention on decreasing the perceived norm of alcohol consumption in glasses per week during course periods – closer to the actual norm – among students from the intervention group. However, the effect is considered small due to a low R-squared of the analysis and should therefore be interpreted with caution. Considering the working mechanism of the SNA, correction of the perceived norm is the mediating step in changing unhealthy behaviour: the framework of Keller and Bauerle implies that a correction of the misperception needs to take place before the actual unhealthy behaviour will decrease [34, 35]. From our subgroup analysis, it became clear that the intervention effect on the perceived norm of alcohol use was significant among females over the intervention group, but not among males. Here, it should be taken into account that the subgroup of males consisted of only 611 students, which was lower than the necessary subjects according to the sample size calculation. Therefore, the power of the subgroup analysis for males might have been too low to detect a significant effect. Nevertheless, a slightly more pronounced misperception of alcohol consumption of peers was seen in females in our intervention group at baseline, as has been demonstrated in previous studies as well [33]. Furthermore, the post-intervention survey showed that our campaign was more seen by females than by males. This could explain why the intervention effect was more obvious among females in our study.

Initially, an ITT approach was chosen to assess the effectiveness of the campaign as a whole, which is more relevant when evaluating an intervention targeting a whole population [21]. However, a PP analysis was performed to test the impact of the intervention on the perceived norm of exposed students specifically. This subgroup analysis showed a more pronounced intervention effect on the perceived norm among students who were exposed to the campaign compared to the overall intervention group. However, also here a smaller sample size (*n* = 566) than the calculated sample size should be taken into account.

Remarkably, the alcohol consumption in glasses per week during course periods of students from both the intervention and control group increased significantly over the study period, although no effect of the intervention on this increase was identified. A possible explanation for this increase in alcohol use could be a significantly lower alcohol consumption by students during the baseline period due to the COVID-19 pandemic and associated lockdown periods at that time (2021). This explanation is supported by the results of the HITC survey of 2017, where the average alcohol consumption of Flemish students was 12.9 glasses per week during course periods [36], compared to the overall average of Flemish students in 2021 of 9.2 glasses per week [7]. Nevertheless, it was decided to not use the HITC survey of 2017 as baseline measurement in this study, because the data would have been more outdated. Furthermore, the use of a control group and DiD approach corrected for this potential COVID-19 effect in our analyses.

Several previous studies also have been unable to identify an intervention effect of an SNA intervention on the alcohol consumption of students. For instance, DeJong et al. (2009) did not find an effect of a large SNA campaign targeting students in a randomised controlled trial, on both the perception of alcohol consumption and alcohol consumption itself [37]. Furthermore, Foxcroft et al. (2015) concluded from their systematic review and meta-analysis of social norms interventions for alcohol misuse in university and college students, that no substantive meaningful benefits were associated with SNA interventions regarding the prevention of alcohol misuse [29]. However, Martens et al. (2013) did show that their SNA intervention was effective in reducing alcohol use and perceived drinking among students [30]. Here, the intervention was carried out as personalised feedback sessions in person. Nevertheless, the effect size was very small, with only a 10% reduction in drinks per week in the intervention group.

There could be various possible interpretations of why our intervention did not decrease the alcohol consumption of students in our study. First, the follow-up period could have been too short. To illustrate, Martens et al. (2013) stated that the effect of their intervention on alcohol use at the six-month follow-up was mediated by changes in perceived norms at the one-month follow-up [30]. Considering this mediation effect of the change in the perceived norm and the fact the intervention in our study affected the perceived norm of alcohol consumption significantly post-intervention, it might be possible that it was too early to detect a significant effect of the intervention on alcohol consumption. Follow-up research could address this issue. Second, the intervention itself might not have had the expected effect on the outcome of alcohol consumption, due to mainly focussing on one determinant of changing health behaviour, namely normative beliefs. Regarding the theory of planned behaviour, attitude and perceived behaviour control are other determinants that play an important role in changing behaviour, which were not targeted specifically in our study [38]. Moreover, the social norm messages that formed the basis of our SNA intervention were general and not tailored to specific subgroups of students. It might be beneficial to adjust the social norm messages to a more specific reference group, for instance by formulating different social norm messages for males and females, or students of different ages. This has been done by Martens et al. (2013), with good results on both the perception of alcohol consumption among peers and alcohol consumption itself [30].

Another possible reason for the lack of an intervention effect on alcohol consumption in our study, and the rather small effect on the perception of alcohol consumption of peers, might be that the implementation of the intervention was not sufficient enough to generate the expected impact. As only 36.3% of the students of the targeted intervention group were exposed to the campaign, we should interpret the outcome of our study in the light of the process evaluation.

Our process evaluation showed that most students saw the campaign via Instagram or Facebook. This is in line with the metrics results of the campaign, which showed a higher reach through Meta than through TikTok. Therefore, we might assume that the respondents of the post-intervention survey form a well-representative group for the process evaluation. However, we do know that the reach of the campaign was bigger than the one-third of University of Antwerp students, as 14.6% of the Ghent University students who filled in the post-intervention survey were also exposed to the campaign. However, because the focus of this study was on the total target population to allow the evaluation of the campaign strategy, rather than the intervention on the individual level, these students were excluded from the study.

The following outcomes of the process evaluation should be considered when evaluating the results of the study. First, campaign metrics data showed a good result for cost/result of €0.10 and €0.13 (for the initial and boost campaign, respectively) per website click among the University of Antwerp advertisements, which was below their target of €0.20. However, the results of the City of Antwerp had a lower effectiveness. Their results on Meta showed a cost/result of €7.25 and €6.10 per reach of 10,000 (for the initial and boost campaign, respectively), which was significantly higher than similar previous campaigns. Furthermore, the campaign resulted in less interaction (likes and reactions) and clicks to the website than expected. Therefore, they concluded that the campaign did not catch on sufficiently with the target group. This is in line with the results of the post-intervention survey, as more than half of the exposed students reported that they did not find the campaign appealing.

However, on the other side, the majority of the exposed students did find the campaign credible and more than half of the students indicated that the campaign has made them aware that students drank less alcohol than they initially thought. Also, 38% of students reflected on their own alcohol consumption due to the campaign. Nevertheless, the campaign might have been more successful when it would have been more appealing to the target group. Therefore, in future research, the impact of an SNA campaign might be larger when the participation of students in the development and implementation process is better embedded. For instance, by making use of focus group discussions with students to assess their needs regarding SNA interventions targeting alcohol and discuss the potential effects of various prototypes of SNA campaigns, instead of consultation of representative students only. Or by letting them engage in the development and design of the campaign materials, to make sure their participation is guaranteed (level 6 of the ladder of participation of Arnstein [18]), which was now only the case for the student vlog.

Second, there might be a chance that the exposure time of the campaign was insufficient to achieve the desired impact. Here, a limitation of SNA interventions in general is that the ideal duration of such interventions has not yet been established. Third, it should be taken into account that a proportion of the University of Antwerp students might not make use of social media and therefore, were not or less exposed to the social media campaign. However, it is known that 94% of 18–24-year-olds in Flanders make use of social media and chat platforms of Meta every day (Facebook, Instagram and WhatsApp), and that Instagram is the most important app for 77% of this age group (44). Therefore, the proportion of University of Antwerp students who do not use social media platforms is probably negligible. Finally, the impact of the intervention on the outcome could be mediated by the extent to which the targeted students were sensitive to social norm messages. In general, students who overestimate the alcohol consumption of their peers, are more susceptible to an SNA intervention, as SNA is based on correcting these misperceptions [17]. In our study, almost a third of the target group did not have misperceptions of alcohol consumption of peers at baseline, and consequently, were less likely to be affected by the intervention.

There were several limitations to the present study. First, we used a quasi-experimental study design. This resulted in a lower quality of evidence compared to randomised controlled trials. However, a quasi-experimental design is a more realistic approach, and therefore, it increased the external validity of our study. Second, not the exact same students participated in the pre and post-measurement. Because this could have led to selection bias, the results of the study need to be interpreted with caution. However, we tried to correct this bias by making use of a DiD approach in our analyses. Third, all data regarding alcohol consumption was obtained retrospectively by self-report assessments. Thus, there is a chance that the results of the study were affected by a self-presentation bias. However, this would have been the case at both baseline and endline and in both the intervention group and control group, and therefore the effect of this bias was probably small. Furthermore, previous research suggested that results of self-report assessments regarding alcohol use are generally reliable and valid [39]. In addition, it might also have been the case that students' responses were influenced by social desirability, which was not assessed in our study. We tried to reduce the chance of this response bias by clearly stating the anonymous character of the surveys.

Despite these limitations, this study made an important contribution to the literature on SNA campaigns regarding alcohol use among students, especially by taking into consideration the common pitfalls of SNA research [14]: by using a baseline measurement, post-intervention measurement and control group, the quality of the study has been improved.

Future research could address several additional issues, for instance, investigating the ideal duration of an SNA campaign and follow-up period and incorporating qualitative analyses for the development and evaluation of the intervention, to get a better insight into facilitators and barriers related to the implementation of the intervention. Furthermore, adjusting social norm messages to more specific reference groups would be of added value. With the help of our thorough process evaluation, we hope to support the implementation of SNA campaigns regarding alcohol use among other universities. In this regard, the outcomes of our process evaluation could contribute to the adaptation and implementation process of future research and therefore, the evaluation of the outcomes of such campaigns in different contexts. Specifically in Flanders, the HITC survey offers opportunities for such campaigns in the future, as this survey is conducted every four years among all Flemish Universities, and therefore, offers a solid foundation for studies on SNA interventions in the future. Furthermore, the next HITC survey, which is planned for 2025, could potentially be used to evaluate the long-term effects of the SNA campaign of the current study.

## Conclusions

This study aimed to evaluate the process and outcome of an SNA intervention on alcohol use among university students. A social norm campaign was developed and implemented by the University of Antwerp and the City of Antwerp and spread via their social media channels. Using a quasi-experimental design, students from the University of Antwerp comprised the target group, while Ghent University students served as the control group. The findings of the study provide support for the efficacy of such a campaign in reducing the misperceptions of alcohol consumption of peers among students. The effect was even stronger among female students and students who were exposed to the campaign. However, no significant effect was found on the alcohol consumption itself. This study made an important contribution to the literature, as it was the first study in Belgium to implement and evaluate an SNA intervention targeting alcohol consumption and the perception of alcohol consumption of peers among university students. By taking into account common pitfalls in SNA research and assessing a thorough process evaluation, this study might strengthen the implementation process of similar SNA campaigns for students in the future.

### Supplementary Information

**Additional file 1. **Directed acyclic graphs of a social norms approach intervention on alcohol use among Flemish university students in 2022-2023.

**Additional file 2. **Results of univariate analyses between covariates and primary and secondary outcomes of a social norms approach intervention on alcohol use among Flemish university students in 2022-2023.

**Additional file 3. **Results of subgroup analyses on the secondary outcome of a social norms approach intervention on alcohol use among Flemish university students in 2022-2023.

## Data Availability

The datasets used and/or analysed during the current study are available from the corresponding author on reasonable request.

## Abbreviations

HED: Heavy episodic drinking
HITC: ‘Head in the Clouds’ study
SNA: Social norms approach
AUDIT-C: Alcohol Use Disorder Identification Test Consumption
DAG: Directed acyclic graph
DiD: Difference-in-Difference
ITT: Intention-to-treat
PP: Per-protocol
NMUPS: Non-medical use of prescription stimulants
VAD: Flemish centre of expertise on alcohol and other drugs
