# Supplementary material for: Process and outcome evaluation of a social norms approach intervention on alcohol use among Flemish university students: a quasi-experimental study
Source: Arch Public Health. 2024 Mar 28;82:45. doi: 10.1186/s13690-024-01265-w (PMC10976709; doi:10.1186/s13690-024-01265-w)
Supplement: Supplementary file 1 — Additional file 1. Directed acyclic graphs of a social norms approach intervention on alcohol use among Flemish university students in 2022-2023. [file 13690_2024_1265_MOESM1_ESM.docx]

**Additional file 1**

Directed acyclic graph (DAGs), made with DAGitty, Copyright © 2007 Free Software Foundation, Inc.


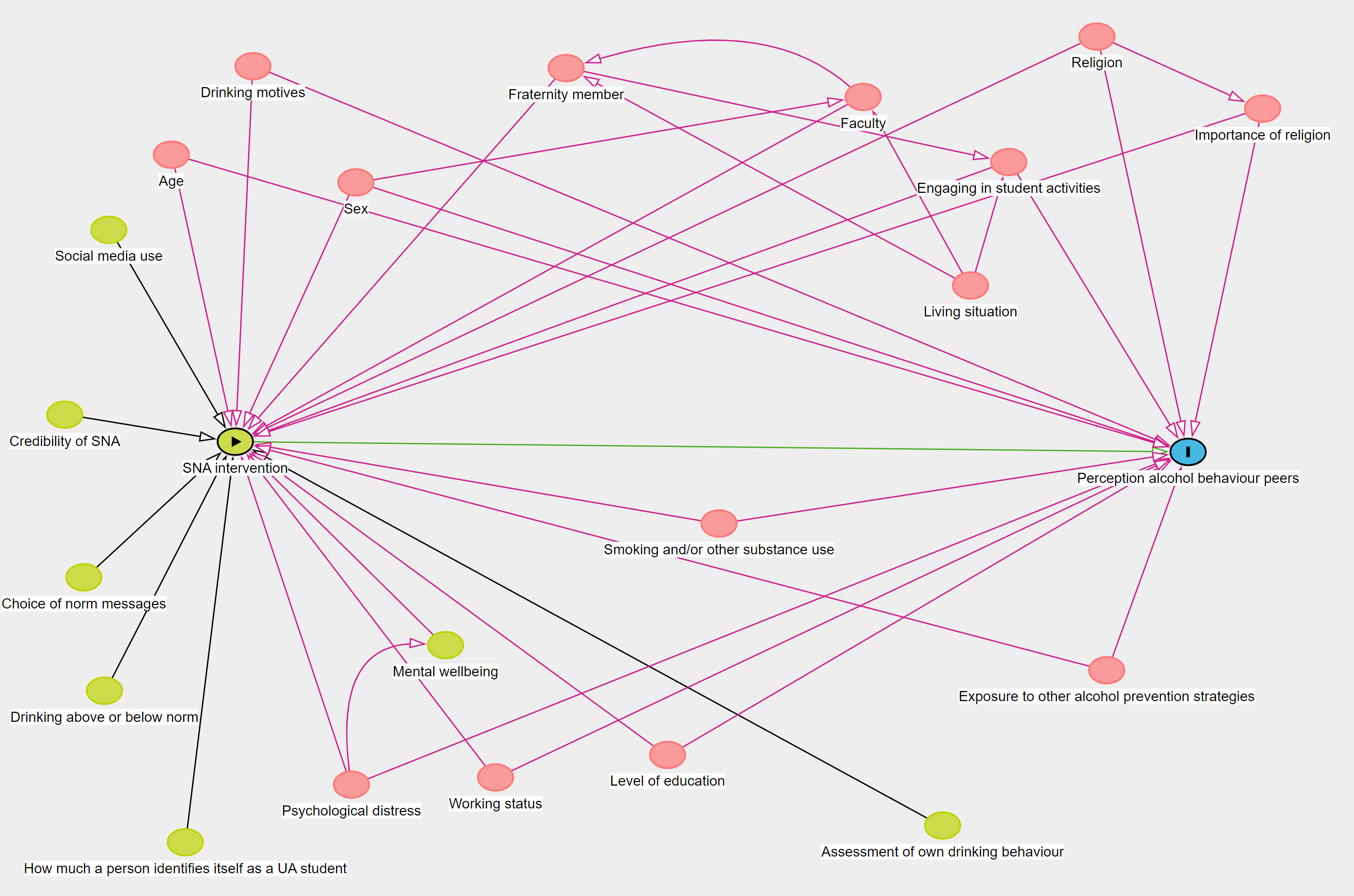


**Figure A1.1:** DAG ‘SNA intervention – perception of alcohol consumption’


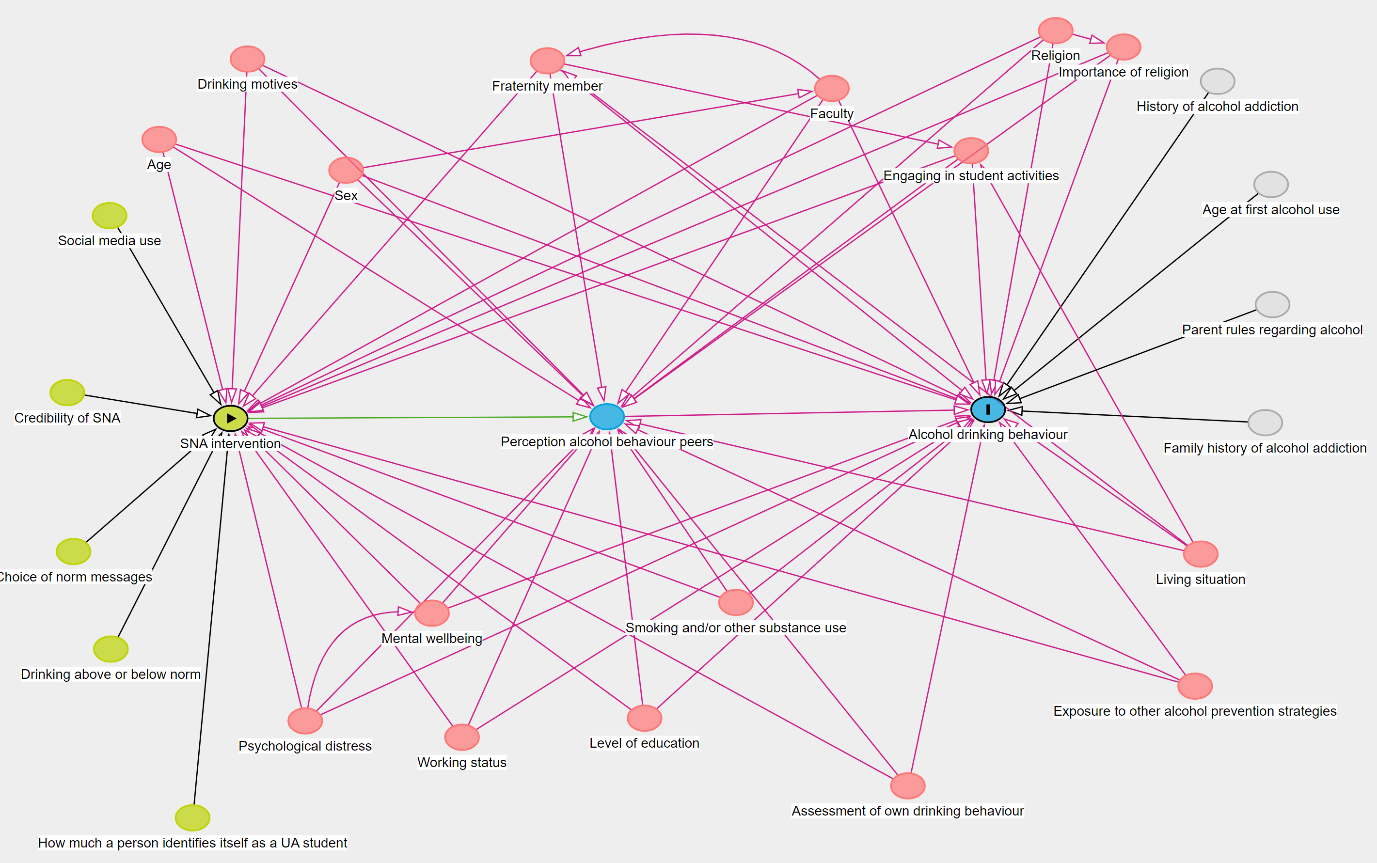


**Figure A1.2:** DAG ‘SNA intervention – alcohol consumption’
