# Supplementary material for: Process and outcome evaluation of a social norms approach intervention on alcohol use among Flemish university students: a quasi-experimental study
Source: Arch Public Health. 2024 Mar 28;82:45. doi: 10.1186/s13690-024-01265-w (PMC10976709; doi:10.1186/s13690-024-01265-w)
Supplement: Supplementary file 2 — Additional file 2. Results of univariate analyses between covariates and primary and secondary outcomes of a social norms approach intervention on alcohol use among Flemish university students in 2022-2023. [file 13690_2024_1265_MOESM2_ESM.docx]

**Additional file 2**

Results of univariate analyses between covariates and primary and secondary outcomes.

| **Table A2.1. Alcohol use of Flemish university students per subgroup, including results of univariate analyses, of a social norms intervention study in 2023** | |  |
| --- | --- | --- |
| **Characteristic** | **Alcohol consumption in glasses per week during course periods, median [IQR]** | **P-value** |
| **Age** |  | 0.004* |
| 17-19 (n = 3222) | 2.3 [8.3] |  |
| 20-21 (n = 3442) | 3.1 [9.6] |  |
| 22-23 (n = 2872) | 3.5 [9.9] |  |
| 24-25 (n = 1002) | 2.8 [7.7] |  |
| **Sex** |  | <.001* |
| Female (n = 6493) | 2.0 [6.4] |  |
| Male (n = 4044) | 5.3 [15.3] |  |
| **Religion** |  | <.001* |
| Christian (n = 3539) | 2.7 [8.4] |  |
| Islamic (n = 261) | 0.0 [0.0] |  |
| No religion (n = 6347) | 3.3 [9.6] |  |
| Other (n = 386) | 2.6 [9.0] |  |
| **Importance of relgion** |  | <.001* |
| Unimportant (n = 6673) | 3.0 [8.6] |  |
| Neutral to important (n = 1822) | 1.5 [6.5] |  |
| **Active fraternity member** |  | <.001* |
| No (n = 8209) | 2.6 [8.5] |  |
| Yes (n = 1513) | 4.8 [13.3] |  |
| **Faculty** |  | <.001* |
| Medicine and Health Sciences (n = 1811) | 2.1 [6.7] |  |
| Veterinary and Pharmaceutical Sciences (n = 953) | 1.5 [5.8] |  |
| Engineering Sciences (n = 1896) | 3.9 [11.5] |  |
| Exact Sciences (n = 874) | 2.1 [8.3] |  |
| Economics (n = 1217) | 5.1 [14.5] |  |
| Political, Social and Educational Sciences and Psychology (n = 1699) | 3.0 [9.3] |  |
| Linguistics and Philosophy (n =1252) | 2.5 [8.3] |  |
| Law and Criminology (n = 823) | 3.4 [9.0] |  |
| **Type of Education** |  | <.001* |
| Bachelor program (n = 6148) | 2.5 [8.8] |  |
| Master program (n=3736) | 3.4 [9.3] |  |
| Bridging program (n = 581) | 3.3 [11.1] |  |
| Other (n = 72) | 3.0 [6.8] |  |
| **Living situation weekdays** |  | <.001* |
| Parental home (n = 4614) | 1.7 [6.5] |  |
| Independently (n = 5916) | 4.1 [11.3] |  |
| **Working status** |  | <.001* |
| Not working (n = 7474) | 2.5 [8.0] |  |
| Working (n =3003) | 4.0 [11.9] |  |
| **Last-year-Cannabis use** |  |  |
| No (n = 6494) | 1.6 [ 5.3] |  |
| Yes (n = 2675) | 8.1 [15.6] |  |
| **Exposure to other campaigns regarding alcohol** |  | 0.017* |
| No (n = 2624) | 4.0 [11.7] |  |
| Yes (n = 114) | 7.3 [13.6] |  |
|  |  |  |
| *Statistically significant |  |  |

| **Table A2.2. Perceived norm of alcohol use of Flemish university students per subgroup, including results of univariate analyses, of a social norms approach intervention study in 2023** | | |
| --- | --- | --- |
| **Characteristic** | **Perceived norm alcohol use (glasses per week in course periods), median [IQR]** | **P-value** |
| **Age** |  | <.001* |
| 17-19 (n = 3593) | 7.0 [7.0] |  |
| 20-21 (n = 3729) | 8.0 [9.0] |  |
| 22-23 (n = 3060) | 10.0 [10.0] |  |
| 24-25 (n = 1102) | 8.0 [9.0] |  |
| **Sex** |  | <.001* |
| Female (n = 7174) | 8.0 [7.0] |  |
| Male (n = 4308) | 10.0 [10.0] |  |
| **Religion** |  | <.001* |
| Christian (n = 3893) | 9.0 [10.0] |  |
| Islamic (n = 276) | 5.0 [5.0] |  |
| No religion (n = 6830) | 8.0 [9.0] |  |
| Other (n = 475) | 8.0 [7.0] |  |
| **Importance of relgion** |  | <.001* |
| Unimportant (n = 7333) | 8.0 [9.0] |  |
| Neutral to important (n = 2061) | 7.0 [7.0] |  |
| **Active fraternity member** |  | <.001* |
| No (n = 9790) | 8.0 [8.0] |  |
| Yes (n = 1658) | 10.0 [9.0] |  |
| **Faculty** |  | <.001* |
| Medicine and Health Sciences (n = 1992) | 8.0 [9.0] |  |
| Veterinary and Pharmaceutical Sciences (n = 1073) | 7.0 [6.0] |  |
| Engineering Sciences (n = 2049) | 9.0 [10.0] |  |
| Exact Sciences (n = 1013) | 7.0 [6.0] |  |
| Economics (n = 1241) | 10.0 [9.0] |  |
| Political, Social and Educational Sciences and Psychology (n = 1830) | 9.0 [9.0] |  |
| Linguistics and Philosophy (n =1429) | 8.0 [7.0] |  |
| Law and Criminology (n = 839) | 9.0 [9.0] |  |
| **Type of Education** |  | <.001* |
| Bachelor program (n = 6773) | 8.0 [7.0] |  |
| Master program (n=4029) | 10.0 [10.0] |  |
| Bridging program (n = 601) | 8.0 [9.0] |  |
| Other (n = 81) | 8.0 [10.0] |  |
| **Living situation weekdays** |  | <.001* |
| Parental home (n = 5366) | 7.0 [8.0] |  |
| Independently (n = 6112) | 10.0 [9.0] |  |
| **Working status** |  | <.001* |
| Not working (n = 8305) | 8.0 [7.0] |  |
| Working (n = 3114) | 10.0 [10.0] |  |
| **Last-year-Cannabis use** |  | <.001* |
| No (n = 8178) | 8.0 [7.0] |  |
| Yes (n = 2826) | 10.0 [9.0] |  |
| **Exposure to other campaigns regarding alcohol** | | 0.106 |
| No (n = 2946) | 9.0 [9.0] |  |
| Yes (n = 128) | 10.0 [10.0] |  |
|  |  |  |
| *Statistically significant |  |  |
