# Supplementary material for: Process and outcome evaluation of a social norms approach intervention on alcohol use among Flemish university students: a quasi-experimental study
Source: Arch Public Health. 2024 Mar 28;82:45. doi: 10.1186/s13690-024-01265-w (PMC10976709; doi:10.1186/s13690-024-01265-w)
Supplement: Supplementary file 3 — Additional file 3. Results of subgroup analyses on the secondary outcome of a social norms approach intervention on alcohol use among Flemish university students in 2022-2023. [file 13690_2024_1265_MOESM3_ESM.docx]

**Additional file 3**

The bootstrapped multiple linear regression analyses on the perceived norm of alcohol consumption for females and males separately are presented in table A3.1 (females) and A3.2 (males).

| **Table A3.1. Results of the bootstrapped multiple regression analysis of a social norms intervention (2023) on perception of alcohol use among female Flemish university students** | | | |  |
| --- | --- | --- | --- | --- |
| **Independent variable** | **B Unstandardized Coefficient** | **β Standardized Coefficient** | **Bootstrapped p-value** | **Bootstrapped 95% Confidence Interval** |
| Group (Intervention) | -0.068 | -0.002 | 0.909 | [-1.288, 1.421] |
| Time (Endline) | 0.139 | 0.003 | 0.718 | [-0.613, 0.861] |
| Group * Time (Intervention * Endline) | -1.643 | -0.028 | 0.045* | [-3.297, -0.222] |
| Fraternity member (Active) | 2.465 | 0.049 | 0.044* | [0.605, 4.782] |
| Living situation weekdays (Independently) | 1.321 | 0.038 | <.001* | [0.625, 1.987] |
| Working status (Working) | 1.086 | 0.028 | 0.031* | [0.156, 2.142] |
| Last-year use of Cannabis (Yes) | 2.419 | 0.057 | 0.02* | [1.225, 3.793] |
|  |  |  |  |  |
| *Statistically significant |  |  |  |  |

| **Table A3.2. Results of bootstrapped multiple regression analysis of a social norms intervention (2023) on perception of alcohol use among male Flemish university students** | | | |  |
| --- | --- | --- | --- | --- |
| **Independent variable** | **B Unstandardized Coefficient** | **β Standardized Coefficient** | **Bootstrapped p-value** | **Bootstrapped 95% Confidence Interval** |
| Group (Intervention) | 0.55 | 0.01 | 0.793 | [-2.100, 3.866] |
| Time (Endline) | -0.963 | -0.017 | 0.132 | [-2.173, 0.161] |
| Group * Time (Intervention * Endline) | -2.354 | -0.029 | 0.158 | [-5.785, 0.363] |
| Last-year use of Cannabis (Yes) | 2.603 | 0.049 | <.001* | [1.083, 4.054] |
|  |  |  |  |  |
| *Statistically significant |  |  |  |  |

Table A3.3 shows the characteristics of students from the intervention group at endline, for exposed students and non-exposed or unknown exposure to the campaign separately.

| **Table A3.3. Characteristics of Flemish university students after running a social norms approach intervention on alcohol use (2023) for exposed and non-exposed students** | |  |
| --- | --- | --- |
| **Characteristic** | **Intervention group** | |
|  | **Exposed** | **Non-exposed or unknown exposure** |
|  | *n* = 556 | *n =* 974 |
| **Sex, n(%)** |  |  |
| Male | 206 (37.1) | 405 (41.6) |
| **Age (years)** |  |  |
| Median [IQR] | 20.0 [3.0] | 20.0 [3.0] |
| **Faculty, n(%)** |  |  |
| Medicine and Health Sciences | 96 (17.3) | 151 (15.5) |
| Veterinary and Pharmaceutical Sciences | 88 (15.8) | 151 (15.5) |
| Engineering Sciences | 56 (10.1) | 172 (17.7) |
| Exact Sciences | 66 (11.9) | 144 (14.8) |
| Economics | 63 (11.4) | 79 (8.1) |
| Political, Social and Educational Sciences and Psychology | 51 (9.2) | 62 (6.4) |
| Linguistics and Philosophy | 87 (15.7) | 131 (13.5) |
| Law and Criminology | 48 (8.6) | 83 (8.5) |
| **Type of education, n(%)** |  |  |
| Bachelor program | 387 (69.6) | 677 (69.6) |
| Master program | 146 (26.3) | 230 (23.6) |
| Bridging program | 20 (3.6) | 62 (6.4) |
| Other | 3 (0.5) | 4 (0.4) |
| **Living situation weekdays, n(%)** |  |  |
| Parental home | 304 (54.8) | 618 (64.9) |
| Independently | 251 (45.2) | 334 (35.1) |
| **Working status** |  |  |
| Working | 220 (39.6) | 387 (39.8) |
| **Religion, n(%)** |  |  |
| Christian | 152 (27.3) | 215 (22.5) |
| Islamic | 17 (3.1) | 42 (4.4) |
| No religion | 363 (65.3) | 654 (68.4) |
| Other | 24 (4.3) | 45 (4.7) |
| **Importance of relgion** |  |  |
| Neutral to important | 81 (42.0) | 135 (45.8) |
| **Active fraternity member, n (%)** |  |  |
| Yes | 175 (31.5) | 130 (13.9) |
| **Last-year-Tobacco use, n(%)** |  |  |
| Yes | 133 (29.6) | 223 (32.8) |
| **Last-year-NMUPS, n(%)** |  |  |
| Yes | 28 (5.0) | 48 (6.4) |
| **Last-year Nonmedical Tranquilizer use, n(%)** | |  |
| Yes | 29 (6.5) | 57 (8.3) |
| **Last-year-Cannabis use, n(%)** |  |  |
| Yes | 121 (26.9) | 164 (24.0) |
| **Ever-use iIlegal drugs, n(%)** |  |  |
| Yes | 54 (12.0) | 118 (17.3) |
| **Life satisfaction (Cantril scale)** |  |  |
| Median [IQR] | 7.0 [2.0] | 7.0 [2.0] |
| **Psychological distress (Kessler-6 scale)** |  |  |
| Median [IQR] | 9.0 [6.0] | 9.0 [7.0] |
| **Exposure to other campaigns regarding alcohol** | |  |
| Yes | 7 (1.4) | 34 (4.5) |
| **Participation in Tournée Minérale (no alcohol for 1 month)** | | |
| Yes | 74 (14.8) | 112 (14.8) |

The results of the bootstrapped multiple linear regression model on the perceived norm of alcohol use among students who were exposed to the campaign (per-protocol subgroup analysis) are shown in Table A3.4.

| **Table A3.4. Results of the bootstrapped multiple regression analysis on perception of alcohol use for Flemish university students who were exposed to a social norms approach intervention on alcohol use (2023)** | | | |  |
| --- | --- | --- | --- | --- |
| **Independent variable** | **B Unstandardized Coefficient** | **β Standardized Coefficient** | **Bootstrapped p-value** | **Bootstrapped 95% Confidence Interval** |
| Group (Intervention) | 0.260 | 0.005 | 0.736 | [-1.021, 1.649] |
| Time (Endline) | -0.264 | -0.005 | 0.432 | [-0.990, 0.344] |
| Group * Time (Intervention * Endline) | -2.147 | -0.021 | 0.008* | [-3.798, -0.538] |
| Sex (Male) | 1.796 | 0.042 | <.001* | [0.872, 2.867] |
| Fraternity member (Active) | 1.644 | 0.028 | 0.019* | [0.361, 3.304] |
| Living situation weekdays (Independently) | 0.984 | 0.023 | 0.014* | [0.234, 1.652] |
| Last-year use of cannabis (Yes) | 1.877 | 0.039 | <.001* | [1.170, 2.610] |
| Ever-use illegal drugs (Yes) | 2.182 | 0.035 | 0.009* | [0.786, 4.051] |
|  |  |  |  |  |
| *Statistically significant |  |  |  |  |
